# The impact of prehospital tranexamic acid on mortality and transfusion requirements: match-pair analysis from the nationwide German TraumaRegister DGU®

**DOI:** 10.1186/s13054-021-03701-7

**Published:** 2021-08-04

**Authors:** Sebastian Imach, Arasch Wafaisade, Rolf Lefering, Andreas Böhmer, Mark Schieren, Victor Suárez, Matthias Fröhlich, TraumaRegister DGU

**Affiliations:** 1 Department of Trauma and Orthopedic Surgery, Cologne-Merheim Medical Center (CMMC), University Witten/Herdecke, Cologne, Germany; 2 Institute for Research in Operative Medicine (IFOM), University Witten/Herdecke, Cologne, Germany; 3 Department of Anaesthesiology and Intensive Care Medicine, Cologne-Merheim Medical Center (CMMC), University Witten/Herdecke, Cologne, Germany; 4 Department II of Internal Medicine (Nephrology, Rheumatology, Diabetes and General Internal Medicine) and Center for Molecular Medicine Cologne, University of Cologne, Faculty of Medicine and University Hospital Cologne, Cologne, Germany; 5 Committee on Emergency Medicine, Intensive Care and Trauma Management (Sektion NIS) of the German Trauma Society (DGU), Berlin, Germany

**Keywords:** Trauma, Bleeding, Coagulopathy, TXA, Tranexamic acid, Hemostatic disorders, Mass transfusion, Trauma care

## Abstract

**Background:**

Outcome data about the use of tranexamic acid (TXA) in civilian patients in mature trauma systems are scarce. The aim of this study was to determine how severely injured patients are affected by the widespread prehospital use of TXA in Germany.

**Methods:**

The international TraumaRegister DGU® was retrospectively analyzed for severely injured patients with risk of bleeding (2015 until 2019) treated with at least one dose of TXA in the prehospital phase (TXA group). These were matched with patients who had not received prehospital TXA (control group), applying propensity score-based matching. Adult patients (≥ 16) admitted to a trauma center in Germany with an Injury Severity Score (ISS) ≥ 9 points were included.

**Results:**

The matching yielded two comparable cohorts (*n* = 2275 in each group), and the mean ISS was 32.4 ± 14.7 in TXA group vs. 32.0 ± 14.5 in control group (*p* = 0.378). Around a third in both groups received one dose of TXA after hospital admission. TXA patients were significantly more transfused (*p* = 0.022), but needed significantly less packed red blood cells (*p* ≤ 0.001) and fresh frozen plasma (*p* = 0.023), when transfused. Massive transfusion rate was significantly lower in the TXA group (5.5% versus 7.2%, *p* = 0.015). Mortality was similar except for early mortality after 6 h (*p* = 0.004) and 12 h (*p* = 0.045). Among non-survivors hemorrhage as leading cause of death was less in the TXA group (3.0% vs. 4.3%, *p* = 0.021). Thromboembolic events were not significantly different between both groups (TXA 6.1%, control 4.9%, *p* = 0.080).

**Conclusion:**

This is the largest civilian study in which the effect of prehospital TXA use in a mature trauma system has been examined. TXA use in severely injured patients was associated with a significantly lower risk of massive transfusion and lower mortality in the early in-hospital treatment period. Due to repetitive administration, a dose-dependent effect of TXA must be discussed.

## Background

Exsanguination still remains the leading, preventable cause of early mortality in trauma patients [1]. Acute traumatic coagulopathy (ATC) aggravates hemorrhage as data already showed over a decade ago [2, 3].

State-of-the-art early resuscitation strategies as proposed by current trauma guidelines aim beside stabilization of oxygenation and perfusion at restoring a physiological clotting capability [4, 5]. Administration of blood products should use fixed, predefined ratios of packed red blood cells (pRBC), plasma and platelets [6, 7]. Hemostatic agents can be used in predefined empiric treatment strategies or guided by goal directed therapy [8–10]. Those strategies have been well adopted in German trauma centers in the last decade [11].

Fibrinogen is the first coagulation factor reaching critical low levels. Decreased fibrinogen levels have been reported in up to 40% of hypotensive trauma patients [3, 12, 13]. Since early hyperfibrinolysis may contribute to this effect, a rapid use of the antifibrinolytic agent tranexamic acid (TXA) is recommended [14, 15]. However, there are common different states of fibrinolysis, i.e., hypofibrinolysis representing an impaired activation of the fibrinolytic system or even fibrinolysis shutdown representing an inhibition beyond a physiologic level after activation of the fibrinolytic system [16]. TXA is associated with increased mortality when given to patients with physiologic levels in fibrinolysis and no benefit in patients in fibrinolysis shutdown [17]. Patients received TXA may even have an increased risk of fibrinolysis shutdown [18].

The fifth edition of the European guideline on management of major bleeding and coagulopathy following trauma recommends TXA to be administered to the trauma patient who is bleeding or at risk of significant hemorrhage as soon as possible and within 3 h after injury at a loading dose of 1 g infused over 10 min, followed by an i.v. infusion of 1 g over 8 h (GoR 1A). Protocols for the management of bleeding patients should consider administration of the first dose of TXA en route to the hospital (GoR 1C) without waiting for results from a viscoelastic assessment (GoR 1B) [4]. The valid German Level-3 guideline on treatment of patients with severe/multiple injuries recommends, for profusely bleeding patients, TXA must be administered as soon as possible, with 1 g over 10 min and then followed as needed with an infusion of 1 g over 8 h (GoR A). For profusely bleeding patients, prehospital administration of TXA can be worthwhile (GoR 0) [19]. In both guidelines, bleeding is not defined by specific values of vital signs like blood pressure.

These guidelines heavily rely on the results of the CRASH-2 trial (Clinical Randomisation of an Antifibrinolytic in Significant Haemorrhage 2). As a large randomized controlled trial, CRASH-2 showed a survival benefit of bleeding patients or patients at risk for bleeding when TXA was administered within the first 3 h after trauma [20, 21]. Due to methodological shortcomings, a limited effect and the research setting in developing countries those results are still questioned. There are only limited data for civilian trauma patients in Europe. Our own research demonstrated a significant higher early survival rate in German helicopter emergency medical services (HEMS) when TXA was already given in the prehospital setting [22]. Nevertheless, evidence for a benefit of the prehospital use of TXA lacks in developed countries until today.

We hypothesized that among severely injured patients at risk of bleeding admitted to trauma centers in Germany, prehospital TXA administration is associated with reduced transfusion requirements and improved survival.

It can therefore contribute to a better understanding of TXA use in civilian trauma patients in developed countries. Germany represents a country where the aforementioned therapy concepts are well established and widely available. For the first time, an analysis of TXA use could be conducted with data sets of the TraumaRegister DGU®, as its use has been specifically recorded in the TraumaRegister DGU® since 2015.

## Methods

### TraumaRegister DGU®

The TraumaRegister DGU® (TR-DGU) of the German Trauma Society (Deutsche Gesellschaft für Unfallchirurgie, DGU) was founded in 1993 [23]. The aim of this multicenter database is a de-identified and standardized documentation of severely injured patients.

Data are collected prospectively in four consecutive time phases from the site of the accident until discharge from hospital: (A) prehospital phase, (B) emergency room (ER) and initial surgery, (C) intensive care unit (ICU/ICM) and (D) discharge. The documentation includes detailed information on demographics, injury pattern, comorbidities, pre- and in-hospital management, course on intensive care unit, relevant laboratory findings including data on transfusion and outcome of each individual. Data of the prehospital phase (A) are derived from the written documentation of emergency service. The inclusion criterion is admission to hospital via ER alive and subsequent ICU/ICM care or death before admission to ICU. Patients dead in the field are not included in the registry.

The infrastructure for documentation, data management and data analysis is provided by AUC—Academy for Trauma Surgery (AUC—Akademie der Unfallchirurgie GmbH), a company affiliated to the German Trauma Society. The scientific leadership is provided by the Committee on Emergency Medicine, IntensiveCare and Trauma Management (Sektion NIS) of the German Trauma Society. Six hundred eight (608) hospitals are grouped in fifty-four (54) regional trauma networks, covering hole Germany apart from a very few regions [24]. The participating hospitals submit their data de-identified into a central database via a web-based application. Scientific data analysis is approved according to a peer review procedure laid down in the publication guideline of TraumaRegister DGU®.

The participating hospitals are primarily located in Germany (91%), but a rising number of hospitals from other countries contribute data as well (at the moment from Austria, Belgium, Finland, Luxembourg, Slovenia, Switzerland, the Netherlands and the United Arab Emirates). Currently, approximately 30,000 cases from more than 650 hospitals are entered into the database per year. Participation in TraumaRegister DGU® is voluntary. For hospitals associated with TraumaNetzwerk DGU®, however, the entry of at least a basic data set is obligatory for reasons of quality assurance [25–28].

The present study is in line with the publication guidelines of the TraumaRegister DGU® and registered as TR-DGU project ID 2017-023 N.

The updated Revised Injury Severity Classification score (RISC II) was used as model for risk of death prediction. The score consists of the following predictors: worst and second-worst injury (AIS severity level), head injury, age, sex, pupil reactivity and size, preinjury health status, blood pressure, acidosis (base deficit), coagulation, hemoglobin and cardiopulmonary resuscitation. Missing values are included as a separate category for every variable [29].

### Study population

**Fig. 1:**
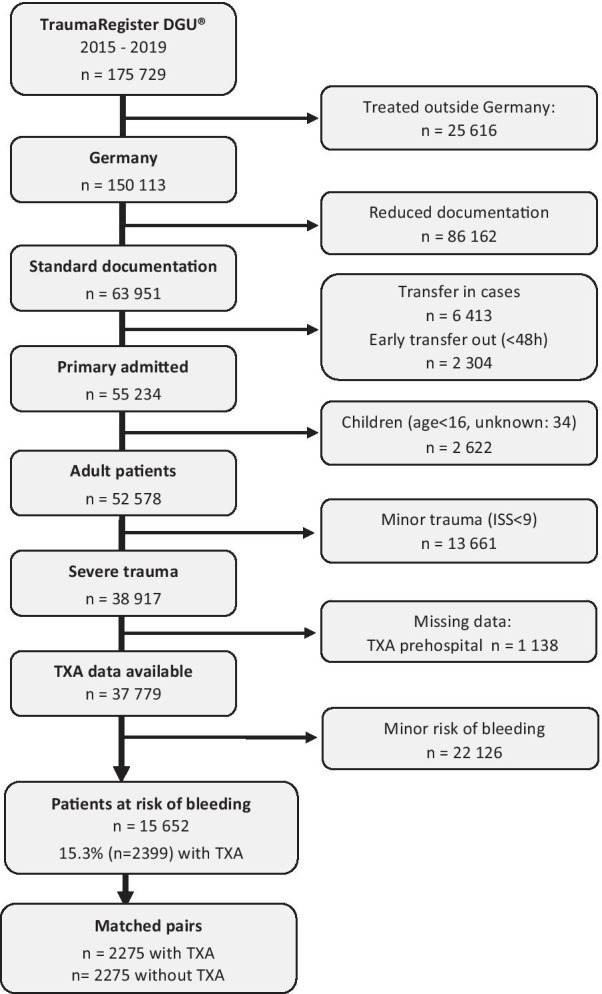
Patient selection flow chart

Since 2015, prehospital administration of TXA is routinely documented for all patients in the TR-DGU. The study included 15,652 severely injured adult patients with risk of bleeding documented from 2015 until 2019. The inclusion criteria were: ISS ≥ 9, age ≥ 16 years, documented TXA use in the prehospital phase, primary admission to a German trauma center. Patients transferred out early (< 48 h) were excluded due to missing final outcome (Fig. 1). Patients documented with the reduced basic data set were excluded as well since no data about TXA use in hospital were available. Patients were considered to be ‘at risk of bleeding’ if at least one of the following criteria was fulfilled: penetrating trauma; hypotension (first prehospital systolic BP < 110 mmHg); or serious injuries (AIS severity ≥ 3) in two or more body regions. Patients defined to be at risk of bleeding received blood transfusion in 20.4% of cases, compared to only 3.3% in patients not at risk of bleeding. Complete data were available for 15,652 patients, of whom 2399 cases (15.6%) received TXA in the prehospital phase (Fig. 1).

A multivariate logistic regression analysis with prehospital administration of TXA as dependent variable was used to create a propensity score. The following variables were included: age; sex; penetrating trauma; severe injury (AIS 3 +) to head, thorax, abdomen and extremities; Glasgow Coma Scale (GCS); systolic BP; prehospital interventions (intubation; catecholamines; thorax drainage); amount of prehospital fluid; mechanism of injury (traffic, high fall); mode of transportation (ground; helicopter); and supra-regional level 1 trauma center as destination.

Based on the propensity score, 2275 cases with prehospital TXA could be matched with 2275 patients who did not receive TXA before hospital admission. Matching was based on an identical propensity score (rounded percentages; exact match) and was performed randomly and blinded to outcome.

The clinical endpoints were requirement of blood transfusion (prevalence, mass transfusions, amount of blood transfused) and mortality (early and in hospital, and due to bleeding). Death of hemorrhage is defined as dying from a trauma-associated bleeding. Mass transfusion was defined as the administration of at least 10 units (250 ml each) of pRBC until ICU admission. Secondary endpoints were volume requirement in the ER, thromboembolic adverse events, length of stay on intensive care unit (ICU) and length of stay in hospital.

### Study approval

The present study was approved by the ethics committee of the Faculty of Medicine, University of Witten/Herdecke, Germany (No. 85/2015).

### Statistical analysis

Statistical analysis was performed using SPSS statistical software (version 24; IBM Inc., Armonk, NY, USA). Data are presented as number of cases with percentage or as mean with standard deviation (SD), respectively. In case of skew distributed data, median with inter-quartile range (IQR) was used instead of means. Group comparisons were made with Chi-squared test in case of categorical data and Mann–Whitney U test for continuous variables. Nonparametric U-test was used for all continuous data irrespective of distribution. For comparison of time to death, Kaplan–Meier survival curves and Mantel–Cox log-rank test was applied. The level of significance was defined at *p* < 0.05; however, due to the large sample size even minor differences may become formally significant, and the large number of test statistics would increase the overall type one error. In addition, we provided 95% confidence intervals (CI) for selected results. Interpretation of results should therefore always consider the clinical relevance of observed differences.

Differences between patients with and without prehospital TXA were also expressed as standardized differences [30]. Results of logistic regression analysis are presented as odds ratios with 95% confidence intervals.

## Results

A total of 15,652 severely injured patients at risk of bleeding were included. The mean age was 52 years, 72% were male, and the mean ISS was 28.7 points. One out of five patients (20.4%) subsequently needed blood transfusion until ICU admission, and 2399 patients (15.3%) received TXA on scene before hospital admission. Basic data of patients with and without prehospital TXA are presented in Table 1.

**Table 1:** Demographic and prehospital characteristics of all patients and of propensity score matched pairs of severe trauma patients with and without tranexamic acid administered during the prehospital phase

	All patients			Matched pairs			Standardized differences all/matched
	TXA *n* = 2399	Control *n* = 13,253	*p*	TXA *n* = 2275	Control *n* = 2275	*p*	
Age (years)	47.4 (19.9)	52.9 (21.0)	< .001	47.6 (19.9)	47.5 (19.9)	.699	0.269/0.005
Male patients (*n*, %)	1777 (74.1%)	9478 (71.5%)	.010	1679 (73.8%)	1688 (74.2%)	.761	0.058/0.009
Penetrating trauma (*n*, %)	221 (9.2%)	1275 (9.6%)	.531	217 (9.5%)	224 (9.8%)	.726	0.014/0.010
ISS (points)	33.0 (15.0)	27.9 (12.8)	< .001	32.4 (14.7)	32.0 (14.5)	.378	0.366/0.027
AIS_HEAD_ ≥ 3 (*n*, %)	1236 (51.5%)	6859 (51.8%)	.834	1161 (51.0%)	1172 (51.5%)	.744	0.006/0.010
AIS_THORAX_ ≥ 3 (*n*, %)	1709 (71.2%)	8897 (67.1%)	< .001	1606 (70.6%)	1636 (71.9%)	.326	0.089/0.029
AIS_ABDOMEN_ ≥ 3 (*n*, %)	642 (26.8%)	2627 (19.8%)	< .001	580 (25.5%)	576 (25.3%)	.892	0.166/0.005
AIS_EXTREMITIES_ ≥ 3 (*n*, %)	1346 (56.1%)	5363 (40.5%)	< .001	1249 (54.9%)	1225 (53.8%)	.475	0.316/0.022
Traffic accident (*n*, %)	1630 (68.3%)	6858 (52.1%)	< .001	1519 (67.1%)	1484 (65.5%)	.262	0.336/0.034
High falls (*n*, %)	405 (17.0%)	2476 (18.8%)	.031	397 (17.5%)	400 (17.7%)	.912	0.047/0.005
Multiple body regions injured (*n*, %)	1948 (81.2%)	9779 (73.8%)	< .001	1837 (80.7%)	1831 (80.5%)	.822	0.178/0.005
BP at scene (mmHg)	108 (40)	118 (38)	< .001	110 (39)	109 (39)	.955	0.256/0.026
Shock (BP ≤ 90 mmHg) (*n*, %)	706 (32.7%)	2500 (21.0%)	< .001	635 (31.0%)	646 (31.4%)	.799	0.266/0.009
First GCS (points)	12 (3–15)	14 (8–15)	< .001	12 (3–15)	11 (3–15)	.212	0.309/0.030
Unconscious (GCS ≤ 8) (*n*, %)	959 (41.0%)	3552 (28.1%)	< .001	888 (40.0%)	911 (41.2%)	.415	0.274/0.024
Helicopter transportation (*n*, %)	1408 (58.7%)	3575 (27.0%)	< .001	1287 (56.6%)	1266 (55.6%)	.530	0.676/0.020
Level 1 trauma center (*n*, %)	2176 (90.7%)	11,098 (83.7%)	< .001	2056 (90.4%)	2044 (89.8%)	.551	0.211/0.020
Intubation (*n*, %)	1660 (69.2%)	5033 (38.0%)	< .001	1538 (67.6%)	1594 (70.1%)	.073	0.659/0.054
Chest tube (*n*, %)	453 (18.9%)	655 (4.9%)	< .001	371 (16.3%)	347 (15.3%)	.329	0.443/0.027
Catecholamines (*n*, %)	844 (36.8%)	2017 (15.2%)	< .001	777 (34.2%)	739 (32.5%)	.232	0.508/0.036
Fluid administration (*n*, %)	2207 (96.8%)	11,188 (92.6%)	.001	2084 (96.7%)	2088 (96.8%)	.731	0.188/0.006
Amount of i.v. fluids (ml)	1000 (1000–2000)	750 (500–1000)	< .001	1000 (900–1700)	1000 (1000–1500)	.266	0.652/0.006
Prehospital time from accident to hospital (min)	78 (29)	69 (32)	< .001	77 (29)	77 (32)	.242	0.295/0.003

Continuous data are presented as mean with standard deviation (SD), except for GCS and fluid volume (median and inter-quartile range); categorical data as number of patients and percent (*n*, %)

ISS, Injury severity Score; GCS, Glasgow Come Scale; BP, systolic blood pressure; i.v., intra venous; AISA, severity according to Abbreviated Injury Scale

A propensity score (= probability to receive TXA in the prehospital phase) was derived from a multivariable logistic model (Table 2). Sex, thoracic injury and treatment in a supra-regional (level 1) trauma center turned out not to be predictive for TXA treatment. A total of 2275 pairs of patients with identical propensity score could be matched. The matched patients with TXA treatment constitute 95% of all patients with TXA treatment.

**Table 2:** Logistic regression model with prehospital administration of tranexamic acid as a dependent variable for generation of propensity score (*n* = 15,652)

Variable	Regression coefficient	OR (95% CI)	*p* Value
Age (ref: < 60 years)			< 0.001
60–69 years	− 0.17	0.84 (0.73–0.97)	0.020
70–79 years	− 0.36	0.70 (0.59–0.82)	< 0.001
80 + years	− 0.42	0.66 (0.54–0.79)	< 0.001
Penetrating trauma	0.52	1.68 (1.40–2.02)	< 0.001
Severe head injury (AIS 3 +)	0.05	1.06 (0.94–1.18)	0.37
Severe abdominal trauma (AIS 3 +)	0.24	1.27 (1.13–1.42)	< 0.001
Severe extremity injuries (AIS 3 +)	0.46	1.58 (1.43–1.75)	< 0.001
Prehospital intubation	0.59	1.80 (1.58–2.05)	< 0.001
Prehospital catecholamines	0.49	1.63 (1.44–1.84)	< 0.001
Chest tube insertion	0.66	1.94 (1.68–2.25)	< 0.001
Glasgow Coma Scale (ref: 14–15)			0.005
9–13	0.09	1.09 (0.95–1.26)	0.22
3–8	− 0.10	0.90 (0.78–1.05)	0.17
Not documented	− 0.41	0.66 (0.49–0.91)	0.010
First systolic BP (ref: > 90 mmHg)			0.011
71–90 mmHg	0.16	1.18 (1.03–1.35)	0.020
1–70 mmHg	0.25	1.29 (1.08–1.54)	< 0.005
not documented	0.03	1.03 (0.86–1.24)	0.75
Prehospital volume (ref: 0–500)			< 0.001
501–1000 ml	0.53	1.70 (1.49–1.94)	< 0.001
1001–2000 ml	0.93	2.54 (2.21–2.92)	< 0.001
> 2000 ml	1.07	2.91 (2.38–3.55)	< 0.001
Not documented	− 0.09	0.92 (0.72–1.17)	0.48
Mechanism: traffic	0.37	1.45 (1.26–1.66)	< 0.001
Mechanism: high fall	0.24	1.28 (1.08–1.58)	0.004
Helicopter transport	0.98	2.66 (2.41–2.94)	< 0.001
Constant	− 3.61		< 0.001

The reference category (ref) is included in brackets behind the variable name. Excluded variables due to minimal effect: male sex (OR 1.00), thoracic trauma (OR 0.99), destination level 1 hospital (OR 1.04)

AIS*,* Abbreviated Injury Scale; BP, blood pressure; n.d.*,* not documented

The two cohorts were well comparable regarding demographic data, injury patterns and prehospital treatment (Table 1). All *p* values were > 0.20, and the standardized differences were < 0.050.

Table 3 shows clinical characteristics upon ER admission, laboratory measures and the extent of interventions (fluids, blood products and hemostatic drugs) in order to compensate hemodynamic instability. Around a quarter of patients in both groups (TXA 24.6%, control 26.4%, *p* = 0.185) have been in shock (i.e., BP ≤ 90 mmHg).

**Table 3:** Clinical characteristics upon hospital admission of matched trauma patients with and without tranexamic acid administered during the prehospital phase

	Tranexamic acid group *n* = 2275	Control group *n* = 2275	*p* value
Shock (BP ≤ 90 mmHg) (*n*, %)	539 (24.6%)	571 (26.4)	.185
BP on admission (mmHg), mean, SD	113 (34)	111 (35)	.108
Hemoglobin (g/dl), mean, SD	11.6 (2.7)	11.7 (2.7)	.305
Base excess (mmol/l) mean, SD	− 4.5 (6.1)	− 4.3 (6.3)	.155
INR mean, SD	1.32 (0.67)	1.32 (0.75)	.094
Fluids (ml) median, IQR	1500 (500–3000)	1000 (500–2150)	< .001
Blood transfusion until ICU (*n*, %)^b^	765 (33.9%)	694 (30.7%)	.022
FFP transfusion until ICU (*n*, %)	462 (20.5%)	435 (19.3%)	.307
Units of pRBC^a^, median, IQR	4 (2–7)	4 (2–9)	< .001
Units of FFP^a^, median, IQR	2 (0–6)	3 (0–7)	.023
Units of pRBC^b^, median, IQR	0 (0–2)	0 (0–2)	.235
Units of FFP^b^, median, IQR	0 (0–0)	0 (0–0)	.501
Hemostatic drugs^c^ (*n*, %)	1034 (47.8%)	939 (44.2%)	.018
TXA in the ER (*n*, %)	749 (32.9)	798 (35.1)	.014

ER, emergency room; pRBC, packed red blood cells; FFP, fresh frozen plasma

^a^Only cases with blood transfusion

^b^Data of all patients

^c^i.e., fibrinogen, prothrombin concentrates (*PPSB*), calcium, factor VII or factor XIII

Patients in the TXA group received significantly more i.v. fluids (median TXA 1500 ml, control 1000 ml, *p* < 0.001). Significant more TXA patients were transfused (33.9% CI 32.0–35.9 versus 30.7%, CI 28.8–32.6, *p* = 0.022) but if transfused, the average amount was significantly lower than in the control group (6.0 units, CI 5.5–6.5 versus 7.6 units, CI 6.9–8.3, *p* < 0.001). The control group also received significantly more FFP (TXA 4.4 units, CI 3.9–4.9, control 5.4 units CI 4.7–6.0, *p* = 0.023). Consequently, the mass transfusion rate was higher in the control group (TXA 5.5% CI 4.5–6.4 versus 7.2% CI 6.2–8.3, *p* = 0.015). Nearly half of all patients in both groups received drugs to treat coagulopathy, but the TXA group receiving significant more (TXA 47.8% CI 45.7–49.9 versus 44.2% CI 42.1–46.3, *p* = 0.018) (Table 3).

About one-third of patients in both groups (TXA 32.9% CI 31.0–34.9, versus 35.1%, CI 33.1–37.0, *p* = 0.014) received TXA after admission in the ER. For TXA patients, this was the second dose, while it was the first dose in control patients.

**Fig. 2:**
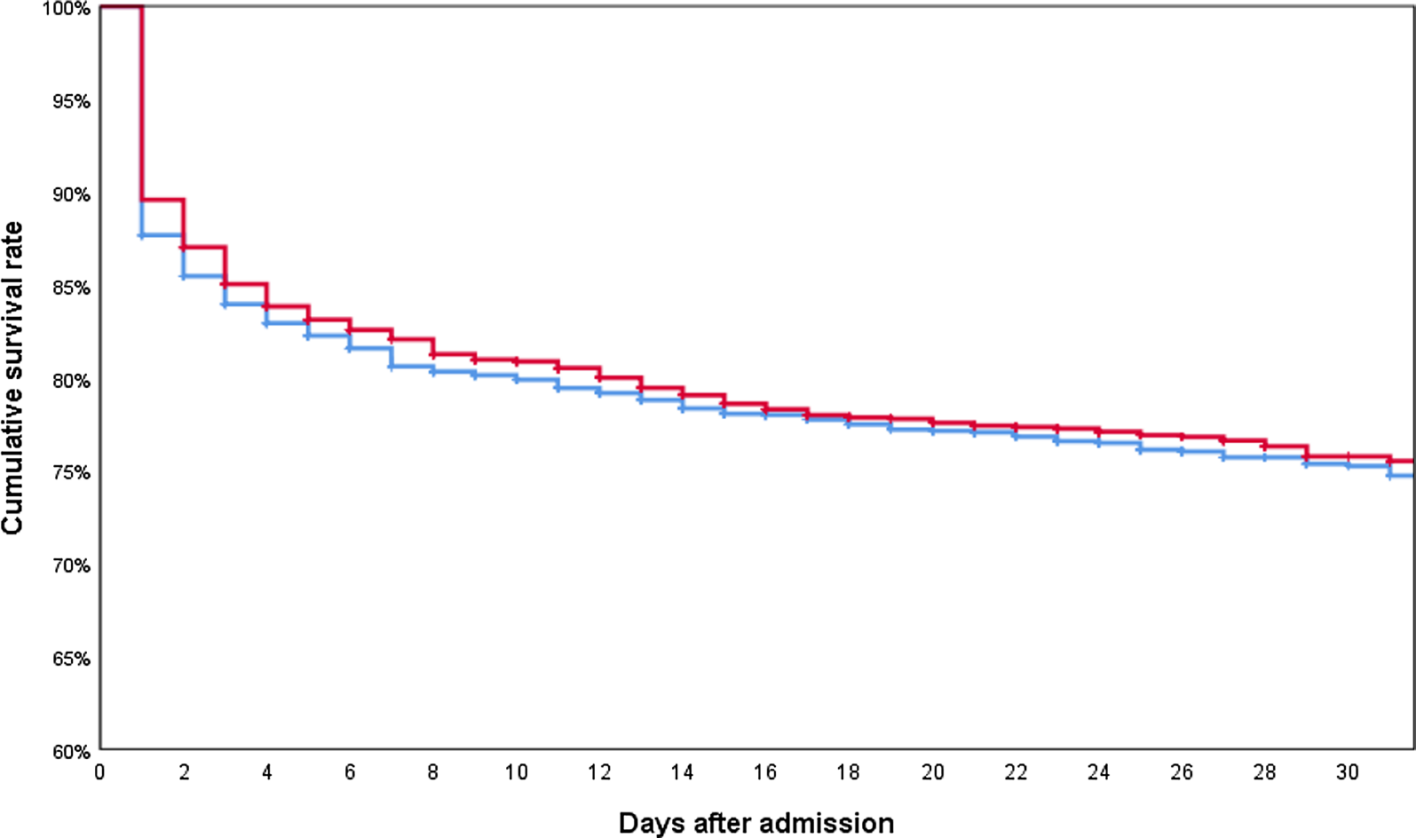
Kaplan–Meier survival rates for the first 30 days following hospital admission for patients with (red line, *n* = 2275) and without (blue line, *n* = 2275) prehospital tranexamic acid. Data were censored in case of discharge or transfer out; *p* = .045 (log-rank test)

Table 4 presents the mortality rates at different time points after hospital admission. There was a highly significant advantage for TXA patients after 6 h hours (6.8% CI 5.8–7.8 versus 9.1% CI 8.0–10.3, *p* = 0.004) but *p* values decreased with increasing time after admission. The 12-h mortality is just significant (*p* = 0.045). This early difference is also visible in the Kaplan–Meier curved (Fig. 2, *p* = 0.045). There is no difference in mortality after 30 days (TXA 22.4% CI 20.7–24.1 versus control 23.0% CI 21.3–24.8 *p* = 0.620).

**Table 4:** Outcome data in matched patients with and without prehospital TXA administration

	Tranexamic acid group	Control group	*p* value
ICU LOS (days) median, IQR	7 (2–18)	7 (2–18)	.125
Hospital LOS (days) median, IQR	19 (8–32)	18 (8–31)	.238
Thromboembolic event (*n*, %)	132 (6.1%)	100 (4.9%)	.080
Sepsis (*n*, %)	277 (13.3%)	249 (12.7%)	.570
Multiple organ failure (*n*, %)	843 (40.0%)	870 (42.6%)	.087
Time to death (days) median, IQR	2 (1–8)	2 (1–6)	.933
6-h mortality (*n*, %)	155 (6.8%)	208 (9.1%)	.004
12-h mortality (*n*, %)	239 (10.5%)	282 (12.4%)	.045
24-h mortality (*n*, %)	297 (13.1%)	333 (14.6%)	.122
30-day mortality (*n*, %)	510 (22.4%)	524 (23.0%)	.620
In-hospital mortality (n, %)	531 (23.3%)	553 (24.3%)	.444
Expected mortality rate based on RISC II prognosis (%)	24.8%	25.1%	.680
Death due to hemorrhage (*n*, %)	68 (3.0)	97 (4.3)	.021

ICU, intensive care unit; LOS, length of stay

In the matched pair collective 1084 of 4550 patients died (23.8%). Around one-third died before the first 6 h after admission and only 8.5 percent (*n* = 92) after 30 days. Death due to hemorrhage was the third frequent cause of death observed in all non-survivors, with head injury and multiple organ failure having higher prevalence. Death due to hemorrhage was significant more frequent in the control group (4.3% of all patients CI 3.4–5.1) than in the TXA group (3.0%, CI 2.3–3.7, *p* = 0.021; Table 4). Different causes of death showed different temporal peaks. Hemorrhage was most common in the first 6 h, while multiple organ failure only developed over time. Traumatic brain injury (TBI) was the leading cause of death in all periods with the highest proportion between 7 and 24 h after admission (Table 5).

**Table 5:** Cause of death at different time points and relative distribution within non-survivors (48 cases without documented cause of death)

Time interval	0–6 h		7–24 h		Day 1–30		Total	
Cause of death	TXA (%) *n* = 143	Control (%) *n* = 189	TXA (%) *n* = 126	Control (%) *n* = 102	TXA (%) *n* = 205	Control (%) *n* = 179	TXA (%) *n* = 489	Control (%) *n* = 493
Hemorrhage	31	39	11	17	3	2	14	20
Head injury	46	41	64	50	55	51	55	47
Organ failure	14	12	20	25	34	41	24	27
Others	10	8	6	9	8	6	8	7

The leading cause of death was documented in 95% of all patients who died in hospital.

In both treatment groups, the observed mortality was close to the expected mortality rate based on RISC II prognostic score: 23.3% versus 24.8% in the TXA group and 24.3% versus 25.1% in the control group (Table 4).

Among the secondary outcome measures, ICU length of stay and days in hospital were nearly identical (Table 4). Thromboembolic events occurred more often in the TXA group (6.1% versus 4.9%) but the difference was not significant (*p* = 0.080). Complications like multiple organ failure was observed slightly less common in the treatment group.

## Discussion

To the best of our knowledge, this is the first study to investigate the nationwide use of TXA in a large cohort of civilian trauma patients in a mature trauma system (Germany). By coherently analyzing the data sets of the first five complete years (2015–2019), since beginning of registration two equally sized groups of 2275 severely injured patients each could be identified. These large sample sizes were identical in terms of demographics and injury severity. The sample sizes of the propensity score-based matched-pairs analysis were up to 8 times larger than prior reported studies [31–33], reaching a fifth of the patients included in CRASH 2 [20]. Therefore, the findings add knowledge to the ambiguous data on prehospital TXA use in developed countries.

Patients who preclinically received at least one dose of TXA required significantly less pRBC units, when transfused, and had a significant lower rate of mass transfusion (≥ 10 pRBC units) until ICU admission.

Early in-hospital mortality (after 6 h und 12 h) was significantly lower in the TXA group with a respective trend for the first 24 h after admission [34]. Death due to trauma associated hemorrhage was significantly less common. TBI was the leading cause of death at all time points. In the CRASH 3 study, TXA only reduces the risk of head injury-related death in patients with mild-to-moderate head injury (GCS 9–15, RR 0·78 [95% CI 0·64–0·95]). In patients with severe head injury (GCS 3–8, RR 0·99 [95% CI 0·91–1·07]), no clear evidence of a reduction was found (*p* value for heterogeneity 0·030) [35]. In both groups of the study, around 40% of patients had a GCS score of 8 or less.

In the clinical course, less multiorgan failures were reported in these patients without significantly improving mortality after 30 days. This is in accordance with the findings in the mature trauma system in London, Great Britain, in 2015 (reduction in MOF [odds ratio (OR) = 0.27, confidence interval (CI): 0.10–0.73, *p* = 0.01] [36]. Evidence from cardiothoracic surgery implies a decrease in the inflammatory response by TXA use in dosing up to 80 mg kg^−1^ [37].

It is worth mentioning that in each group around 30 percent of the patients preclinically had at least one episode of systolic blood pressure below 90 mmHg. This sign of hemodynamic derangement was one of the major inclusion criteria of the widely appreciated randomized controlled CRASH-2 trail which showed a reduced all-cause mortality by TXA use (14.5% vs 16.0%; RR 0.91; 95% CI 0.85–0.97; *p* = 0.0035). Larger effects were detected in even more hemodynamically unstable patients, as a 4.5 percent absolute reduction in mortality was achieved among patients presenting with a systolic blood pressure of less than 75 mmHg [21]. The recently published STAAMP-trail (NCT02086500) only demonstrated a lower 30-day mortality for patients with severe shock (SBP ≤ 70 mmHg) when receiving TXA (18.5% vs 35.5%; 95% CI: − 25.8% to − 8.1%, *p* < 0.003) [38]. Current guidelines in Germany, Europe and the USA define a potential risk of bleeding based on CRASH-2 as an indication for TXA use [4, 19]. The randomized PATCH-study (Pre-hospital Anti-fibrinolytics for Traumatic Coagulopathy and Hemorrhage, NCT02187120, 1316 patients planned, estimated completion 01/2021, in 06/2021 still recruiting) tries to objectify the indication of TXA by applying a score (COAST score[39]) [40].

A critical appraisal of the indication for TXA use seems mandatory since Moore et al. showed that only the smaller part of patients effectively has hyperfibrinolysis (18%, *n* = 33/180) 30 min after admission while the majority was in shutdown (64%, *n* = 115/180). However, the hyperfibrinolysis group showed a higher mortality (44% to 17%) [14, 18].

In our present study, slightly more than 30 percent of patients in the TXA group received a second dose of TXA after admission to the hospital in ER (32.9%, *n* = 749/2275). That approach represents common practice and corresponds to the approach in the control group. Lier et al. discussed a weight-dependent administration of TXA in comparison with a regime based on empirical CRASH-2 dosing (1 g i.v. TXA over 10 min, followed by 1 g i.v. TXA over 8 h) [41, 42]. A Swiss study described that in 21 percent a single prehospital i.v. dose of 1 g TXA fails to reach adequate plasma levels (> 20 µg/ml) at hospital admission (71 patients, mean plasma level TXA 28.7 μg/ml, IQR 21.5–38.5, range 8.7–89.0, mean ISS 20) [43]. Using different dosing regimen, the aforementioned STAAMP-trail revealed a survival benefit in those patients who received a repeat bolus of TXA (3 g TXA in total; 2 times 1 g as bolus infusions in 10 min and 1 g during the following 8 h) [38]. The placebo-controlled TAMPITI-study also investigates a different dosing regimen (2 g vs. 4 g i.v. TXA, Tranexamic Acid Mechanisms and Pharmacokinetics in Traumatic Injury, NCT02535949, 150 patients, estimated completion 12/2018, no updates since then).

Around a quarter of patients in the control group reached ER in shock without prehospitally receiving TXA. This might indicate that widespread use of TXA has not been reached in the mature trauma system of Germany yet, since a relevant proportion of patients might not have received the medication formally indicated.

A relevant portion of patient in the control group received a first dose of TXA after ER admission (control 35.1.9%, TXA 32.9%). Taking the duration of the prehospital phase into account (control mean 77 ± 32 min), most of the patients were within in the cutoff for higher survival defined by CRASH-2 (survival benefit < 3 h). At the same time, CRASH-2, reinforced by data of CRASH-3, describes a dynamic time-to-treatment relationship favoring an early potentially prehospital administration [20, 21, 35].

There was increased risk of thromboembolism in the TXA group as potential risk of TXA use, like reported in the MATTERS-study and more recently in the HALT-IT-trial, where actually a higher dosage was used [44–46].

Several limitations of the present study must be addressed since data are derived from a registry. Unlike in a prospective study, not every possible outcome-relevant variable can be controlled. The propensity matching in this study can therefore only show an association of the prehospital TXA administration with the observed effects in the groups. A direct causation cannot be shown for methodological reasons. The effects could, for example, also result from differences in clinical treatment standards between the trauma centers involved.

The available data in the registry also have limitations.

First, laboratory parameters with respect to hyperfibrinolysis or inflammation (e.g., D-dimers, interleukin-6) are not available in the database. Viscoelastic testing which can reveal the coagulopathic state is not specifically documented. Therefore, it remains unclear how many patients effectively have been in a hyperfibrinolytic state. Second, the documentation of data was partly incomplete and inconsistent with respect to morbidity (organ failure, sepsis, thromboembolism). There is some further limitation because exact timing of prehospital TXA administration and dosages of TXA have not been documented. It can be assumed that dosage mainly followed current guidelines (1 g i.v. TXA en route to hospital) but there is no nationwide standard (SOP) and at the discretion of the emergency physician.

Some prescribed potential risks of TXA administration like seizures or renal necrosis have not been specifically documented in the registry [47–49].

Patients, having received TXA preclinically, seem to trigger a more comprehensive resuscitation approach in the early hospital phase since TXA group received more i.v. fluids, blood transfusion were started more frequently and more hemostatic drugs were administrated. However, no difference could be found between the groups in terms of laboratory data and vital signs at admission to the hospital. The suspected risk of bleeding can be an explanation for this.

At the European level, it is recommended for patient safety to bundle those measures in local treatment protocols [50]. Blood products and hemostatic drugs (i.e., calcium, fibrinogen) are part of those bundles in the initial resuscitation phase before goal directed therapy is started [4, 33, 51]. The same approach could be found in a relevant proportion of patients in the control group.

## Conclusion

In the present study in a mature, civilian trauma system TXA administration was associated with a significant lower amount of pRBC units when transfused and a significantly lower rate of mass transfusion. The early in-hospital mortality after 6 h and 12 h was significantly lower, and death to hemorrhage was reported significantly less frequently in the TXA group.

Further prospective studies should clarify lack of evidence for exact dosage and precise indication [40, 52].

## Data Availability

The data set used and analyzed during the current study is not available due to data protection guidelines of the German Trauma Society.
